# Gemcitabine-Induced Extensive Skin Necrosis

**DOI:** 10.1155/2012/831616

**Published:** 2012-11-26

**Authors:** Sara D'epiro, Monica Salvi, Carlo Mattozzi, Simona Giancristoforo, Marco Campoli, Ramona Zanniello, Cecilia Luci, Laura Macaluso, Sara Giovannoni, Roberto Iacovelli, Stefano Calvieri, Antonio Giovanni Richetta

**Affiliations:** ^1^Dipartimento di Dermatologia, Policlinico Umberto I, “Sapienza” Università di Roma, Viale del Policlinico 155, 00160 Roma, Italy; ^2^Dipartimento di Scienze Radiologiche, Oncologiche e Anatomo-Patologiche Policlinico Umberto I, “Sapienza” Università di Roma, Viale del Policlinico 155, 00160 Roma, Italy

## Abstract

An 82-year-old woman presented with oedema and extensive necrotic ulcerative lesions on the back side of her lower limbs, emerging after the second cycle of chemotherapy consisting of Gemcitabine for metastatic pancreatic cancer. The absence of any convincing argument in favor of cardiovascular or autoimmune disease led us to attribute the onset of skin necrosis to chemotherapy administration. Although skin ischemia has also been described as a paraneoplastic syndrome, in this case we could observe a temporal and causal relationship to Gemcitabine infusion. Recently, this drug has been associated with important vascular side effects; its vascular toxicity is in fact higher than previously estimated. To our knowledge, careful attention should be reserved to neoplastic patients candidated to Gemcitabine administration, especially if previously affected by arterial vascular disease, venous thromboembolism, or collagenoses.

## 1. Introduction 

Cutaneous side effects are relatively frequent events related to chemotherapy administration.

They are commonly observed during cancer therapy with EGFR inhibitors, which include acneiform eruptions, paronychia, xerosis, fissures, hyperpigmentation, alterations in hair growth, and telangiectasia [1]. 

Several chemotherapy-induced skin reactions have been described in literature, including hypersensitivity, nail pigmentation, onycholysis, rash, toxic epidermal necrolysis, scleroderma, and palmar-plantar erythrodysesthesia (PPE). 

PPE is a common side effect due to cytotoxic chemotherapy, mostly related to 5-fluorauracil (5-FU), capecitabine, liposomial-encapsulated doxorubicin, and cytarabine. 

It includes symptoms such as painful swelling of the hands and feet with initial paraesthesia, followed by erythema and later by desquamation, which may progress to ulceration [2]. 

As far as Gemcitabine administration, cutaneous toxicity is reported in approximately 25% of patients, being skin manifestations mostly of a mild grade (WHO G1-G2) [3]. 

The etiology of cutaneous reactions remains unclear and its treatment is symptomatic. Most of the toxicity is reversible with chemotherapy dose reduction or delay [3]. 

## 2. Case Report 

**Figure 1:**
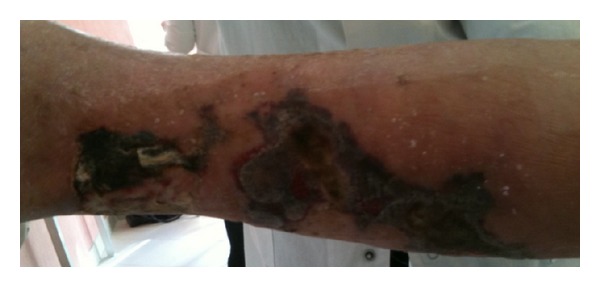
Extensive skin necrosis of the lower limb.

An 82-year-old woman was admitted to our department for the presence of oedema and extensive necrotic ulcerative lesions on the back side of her lower limbs (Figure 1).

The patient reported a 20 days history of severe pain and paraesthesia of lower limbs, emerged after the second cycle of chemotherapy consisting of Gemcitabine for pancreatic carcinoma with lung and liver metastases. The dosage administrated was 1000 mg/mq every 7 days for three times each cycle, with a resulting total dose of 10.8 gr. 

The previous medical history was unremarkable for cardiovascular risk factor or underlying vascular disease. 

She was not taking any kind of drugs or anticoagulant therapy.

The patient was afebrile, while laboratory test revealed a mild increase of inflammatory markers and a mild anaemia (C-reactive protein 6.8 ng/dL, leukocytosis 12.6 × 10³/*μ*L, and haemoglobin 9.5 g/dL). Autoimmunity screen was negative and coagulation parameters were in the normal range (p,c-ANCA, ANA, ENA, anti-DNA, C3 and C4 complement, cryoglobulins, cardiolipin antibodies, fibrinogen, INR). 

A swab taken from the lesions was not suggestive for skin infection. 

An arterial and venous Doppler ultrasound study of the lower limbs was normal. 

A skin biopsy was not performed, due to the impaired systemic conditions of our patient and her disagreement. 

**Figure 2:**
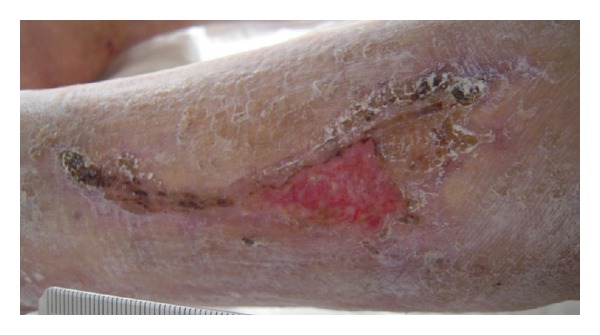
Skin ulcers progressively shrinkaged toward healing after 40 days of withdrawn therapy.

Furthermore, owing to the appearance of the extensive skin necrosis in conjunction with Gemcitabine infusion and metastases progression, the patient discontinued chemotherapy and introduced advanced local wound care with hydrogel and polyurethane materials. Skin ulcers progressively shrinkaged toward healing in 40 days (Figure 2). The patient died three months later owing to spreading metastases. 

## 3. Discussion 

The absence of any convincing argument in favor of cardiovascular or autoimmune disease led us to attribute the onset of skin necrosis to Gemcitabine therapy. Although skin ischemia has also been described as a paraneoplastic syndrome, in this case we could observe a temporal and causal relationship to Gemcitabine administration. Furthermore, paraneoplastic gangrene usually appear through acral ischemia and digital necrosis with Raynaud's syndrome and acrocyanosis [4]. Finally, the progressive healing observed after the interruption of chemotherapy could not be achieved if it was a paraneoplastic syndrome. 

Gemcitabine is an antimetabolite nucleoside employed in the treatment of carcinoma of the lung, pancreas, and urothelium. It is usually well tolerated, while the major side effects reported are myelotoxicity, skin rashes, influenza-like syndrome, and rarely severe pulmonary toxicity with interstitial lung disease [5, 6]. 

Cutaneous toxicity is reported in approximately 25% of patients, including alopecia, macula-papular eruption, urticaria, palmar-plantar erythrodysesthesia, and acute lipodermatosclerosis-like reactions. However, these conditions are predominantly mild (WHO-grade 3-4 <1%) and not limited to special regions of the body like hands or feet. The skin toxicity described is often a transient rash which tends to be macular and not occurring with every course of therapy [7]. 

Recently, Gemcitabine has been associated with important vascular side effects; its vascular toxicity is in fact higher than previously estimated. Acute arterial events and venous thromboembolism, digital ischemia and necrosis, vasculitis and thrombotic microangiopathy, potentially fatal systemic capillary leak and reversible posterior leukoencephalopathy syndromes are only a few items on the long list of vascular-toxic effects of Gemcitabine [7]. Voorburg et al. reported a case of Gemcitabine induced vasculitis, re-occurred during both the first and second cycle of chemotherapy, and completely disappeared with the withdrawal of the drug [8]. Ramsay et al. reported a case of large vessel vasculitis presenting as fever of unknown origin, developed during the first and second cycle of chemotherapy; aortic vasculitis reverted with the interruption of the drug and corticosteroid therapy [9]. Vénat-Bouvet et al. reported two cases of thrombotic microangiopathy and digital necrosis due to Gemcitabine administration. Also in these cases symptoms resolved after stopping the chemotherapy, in spite of the progression of the neoplastic disease. Good progresses were obtained when given an intravenously infusion of Ilomedine together with chemotherapy withdrawn [10]. 

However the pathogenesis of Gemcitabine induced vascular toxicity remains unclear. Endothelial damage, increased adherence of platelets, deposition of immune complexes as immunoallergic effects, and a hypercoagulable state may culminate in arterial thrombosis [11]. This kind of adverse event is often underestimated since the diagnosis may be incorrect and the life expectancy of patients is short. In these cases the withdrawal of Gemcitabine therapy is usually recommended [10]. In our case we could refer the etiology of skin necrosis to Gemcitabine therapy owing to the absence of any convincing argument in favor of cardiovascular or autoimmune disease, for the presence of a temporal relationship to Gemcitabine administration, and finally, owing to the progressive healing observed after the interruption of chemotherapy which could not be achieved if it was a paraneoplastic syndrome. 

## 4. Conclusions 

To our knowledge, careful attention should be reserved to neoplastic patients candidated to Gemcitabine administration, especially if previously affected by arterial vascular disease, venous thromboembolism, or collagenoses. Finally, such side effect should always be considered as differential diagnosis for skin ulcers in neoplastic patients.

## References

[1] Ehmann LM, Ruzicka T, Wollenberg A *Management of Cutaneous Side-Effects of EGFR Inhibitors: Conclusion*.

[2] Laack E, Mende T, Knuffmann C, Hossfeld DK (2001). Hand-foot syndrome associated with short infusions of combination chemotherapy with gemcitabine and vinorelbine. *Annals of Oncology*.

[3] Green MR (1996). Gemcitabine safety overview. *Seminars in Oncology*.

[4] Buch RS, Geisbüsch R, Kunkel M (2002). Acral ischemia as a rare paraneoplastic syndrome in the terminal phase of mouth floor carcinoma. *Mund-, Kiefer- und Gesichtschirurgie*.

[5] Marruchella A, Fiorenzano G, Merizzi A, Rossi G, Chiodera PL (1998). Diffuse alveolar damage in a patient treated with gemcitabine. *European Respiratory Journal*.

[6] Pavlakis N, Bell DR, Millward MJ, Levi JA (1997). Fatal pulmonary toxicity resulting from treatment with gemcitabine. *Cancer*.

[7] Chu CY, Yang CH, Chiu HC (2001). Gemcitabine-induced acute lipodermatosclerosis-like reaction. *Acta Dermato-Venereologica*.

[8] Voorburg AMCJ, van Beek FT, Slee PHTJ, Seldenrijk CA, Schramel FMNH (2002). Vasculitis due to gemcitabine. *Lung Cancer*.

[9] Ramsay LB, Stany MP, Edison JD, Bernstein SA, Schlegal KE, Hamilton CA (2010). Gemcitabine-associated large vessel vasculitis presenting as fever of unknown origin. *Journal of Clinical Rheumatology*.

[10] Vénat-Bouvet L, Ly K, Szelag JC (2003). Thrombotic microangiopathy and digital necrosis: two unrecognized toxicities of gemcitabine. *Anti-Cancer Drugs*.

[11] Dasanu CA (2008). Gemcitabine: vascular toxicity and prothrombotic potential. *Expert Opinion on Drug Safety*.

